# Cryoprotective Effects and Quality Maintenance of Antifreeze Proteins and Peptides on Aquatic Products: A Review

**DOI:** 10.3390/foods13060917

**Published:** 2024-03-18

**Authors:** Xinru Fan, Wenhao Geng, Meng Li, Zixuan Wu, Yongsheng Ma, Zhibo Li, Soottawat Benjakul, Qiancheng Zhao

**Affiliations:** 1 College of Food Science and Engineering, Dalian Ocean University, Dalian 116023, China; fanxinru@dlou.edu.cn (X.F.); gengwenhao2024@163.com (W.G.); limeng@dlou.edu.cn (M.L.); wuzixuan@dlou.edu.cn (Z.W.); mayo@dlou.edu.cn (Y.M.); lzb@dlou.edu.cn (Z.L.); 2 Dalian Key Laboratory of Marine Bioactive Substances Development and High Value Utilization, Dalian 116023, China; 3 Liaoning Provincial Marine Healthy Food Engineering Research Centre, Dalian 116023, China; 4 International Center of Excellence in Seafood Science and Innovation, Faculty of Agro-Industry, Prince of Songkla University, Hat Yai 90110, Songkhla, Thailand; soottawat.b@psu.ac.th

**Keywords:** antifreeze proteins and peptides, freezing protection, aquatic products, myofibrillar protein

## Abstract

Aquatic products are gaining popularity due to their delicacy and high nutrient value. However, they are perishable, with a short shelf-life. Frozen storage is associated with adverse effects, leading to protein oxidation and degradation, thereby altering the protein’s structural integrity and subsequently influencing the palatability of protein-based food products. To address these challenges, novel antifreeze peptides have gained significant attention. Antifreeze peptides are a class of small molecular weight proteins or protein hydrolysates that offer protection to organisms in frozen or sub-frozen environments. They offer distinct advantages over conventional commercial antifreeze agents and natural antifreeze proteins. This review provides an overview of the current state of research on antifreeze agents, elucidates their characteristics and mechanisms, and examines their applications in aquatic products. Furthermore, the article offers insights into the prospective development and application prospects of antifreeze peptides.

## 1. Introduction

Freezing is known as a commonly employed method to preserve aquatic products for long-term storage [1]. Myofibrillar proteins (MPs), constitute the major muscle proteins in aquatic animals. They are known to exhibit several functional properties, especially gelation. Nevertheless, they are vulnerable to denaturation, aggregation, and oxidative deterioration during frozen storage. During frozen storage, physicochemical transformations within the protein structure occur. These changes encompass molecular cleavage, cross-links, aggregation, diminishment of total free sulfhydryl groups, and the generation of compounds containing carbonyl groups [2,3]. The structure of MPs is inherently associated with water molecules. The freezing process induces the formation of ice crystals, thus leading to mechanical impairment of MPs, particularly protein denaturation [3]. In addition, during transportation or sale, multiple rounds of freeze–thawing can result in protein oxidation and denaturation. These phenomena are associated with polymerization between amino acid side chains, perturbing the peptide backbone and culminating in the formation of disulfide bonds [4,5]. Temperature fluctuations during cold chain transportation can cause small ice crystals to recrystallize and further form larger particles. This in turn adversely impacts the original structural integrity of food, causing nutritional loss and a concomitant reduction in the quality of frozen food [6]. Therefore, to ensure the quality of protein-based food during frozen storage, the effective control of ice crystal growth and recrystallization is a promising approach.

Nowadays, the incorporation of cryoprotective antifreeze agents has emerged as an effective strategy to mitigate the quality loss of frozen aquatic products throughout the cold chain. Traditional commercial antifreeze agents are predominantly composed of polyphosphates, sugars, alcohols, and their combinations. Phosphates, due to their water retention enhancement, pH adjustment, and increased affinity with calcium and magnesium ions, are capable of imparting antifreeze effects. However, it is noteworthy that excessive phosphate consumption can detrimentally impact calcium absorption and disturb the calcium-to-phosphorus ratio in the body, thereby contributing to osteoporosis and exacerbating cardiovascular and pediatric diseases [7]. Furthermore, the addition of sugars, such as an antifreeze adjust, can bring about health hazards, including metabolic syndrome, obesity, tooth decay, and diabetes. However, the formation of ice crystal particles can be markedly reduced by high concentrations of sugar alcohols or polyphosphates during freezing and frozen storage. As a consequence, there is a continuous pursuit of novel products to overcome the limitations of existing commercial antifreeze agents. Presently, amino acids, specifically L-arginine and L-lysine, along with polysaccharide/oligosaccharide compounds like trehalose, carrageenan, konjac glucomannan, chitosan, and alginate, have been introduced as alternatives. These compounds are able to inhibit ice crystal growth through adsorption and integration at the ice interface. In addition, those compounds can enhance the gel properties and maintain the microstructure of MPs through hydrogen bonding and electrostatic interactions. Consequently, a reduced ice crystal growth rate and a transformation in the morphology of developing ice crystals can be achieved [7,8].

Antifreeze proteins (AFPs) are typically naturally occurring substances synthesized by organisms to withstand cold environmental conditions and are commonly referred to as ice structural proteins [9]. AFPs have the ability to adsorb onto the surfaces of ice crystals, thereby impeding ice crystal growth, restraining ice crystal recrystallization, and altering the morphology of ice crystals. This mechanism is often termed the ‘adsorption-inhibition’ mechanism [6,10]. Nevertheless, the widespread application of AFPs in the food industry has been hampered by the limited quantities of AFPs that can be obtained through natural extraction and tedious purification [11]. As a consequence, there has been growing research focusing on antifreeze peptides devised from food proteins, particularly from food processing leftovers. This review focuses on the impact of antifreeze proteins/peptides on the structural alterations, functional attributes, and interaction mechanisms of products subjected to repeated freeze–thawing cycles and extended frozen storage. This review can serve as valuable information on antifreeze proteins/peptides, offering substantial significance for the advancement of the aquatic product industry.

## 2. Origin of Antifreeze Proteins and Antifreeze Peptides

### 2.1. Antifreeze Proteins (AFPs)

AFPs primarily originate from organisms inhabiting harsh environments, including high-altitude and cold regions. They can be categorized into four groups based on their sources including bacteria, plants, insects, and fish. Notably, *Moraxella* sp. was the first bacterium identified for its production of AFP with low thermal hysteresis activity [12]. Subsequently, various bacteria including *Flavobacterium*, *Chryseobacterium, Pseudomonas,* and *Marinomonas* have been identified as potential producers of AFPs with varying molecular weights [9]. AFPs have also been found in different plants and arthropod species. Ice-binding proteins or ice-structural proteins isolated from grass (*Lolium perenne*) effectively inhibited ice recrystallization to prevent freezing damage [13]. AFPs can be classified through a sequence of isolation, purification, and identification processes. There are six AFPs of antifreeze glycoproteins (AFPG) and four distinct types of antifreeze proteins (AFP I, II, III, and IV), as well as hyperactive AFP [14]. The molecular structure of AFPs generally has a specific amino acid sequence and length, such as a Gly-Pro/Hyp-x tripeptide repeat sequence, GTPG-, and GPP (OH) G-, with a molecular weight less than 2000 Da [15]. Despite the substantial variations in molecular weight, structure, and physicochemical properties among AFPs from different sources, they share common functional properties. They have the ability to bind with ice, thereby inhibiting its growth, exhibiting thermal hysteresis activity, and suppressing ice recrystallization [16]. However, natural antifreeze proteins are found at low concentrations within living organisms. As a result, high purification costs and significant activity loss during the purification process are of major concern. These constraints impede the large-scale production and application of antifreeze proteins.

### 2.2. Antifreeze Peptides (AFPTs)

AFPTs are primarily derived from food proteins from different sources and can be efficiently prepared through specific enzyme digestion [15]. Initial new materials include meat/fish processing leftovers, such as animal/fish skin and others [17,18,19]. The critical proteases/enzymes responsible for the origin of *Bacillus subtilis* and *Aspergillus oryzae*, alcalase, flavourzyme, as well as papain, were subjected to enzymatic hydrolysis [20]. Because of the low hydroxyproline content and weak thermal stability of fish collagen, the physicochemical characteristics of hydrolyzed peptides and their ability to suppress ice crystal growth were considerably different from those of other mammals, such as bovine skin, even though the identical enzymatic hydrolysis conditions were applied [18]. Nonetheless, in the representative feature of hydrolyzed peptides from different sources of collagen/gelatin, there existed a specific amino acid sequence of tripeptides as mentioned in the AFP molecular structure [21,22]. Furthermore, a unique tetrapeptide, e.g., Pro-Ala-Gly-Tyr, was recognized from Amur sturgeon skin as a gelatin-derived cryoprotective agent to postpone fish mince denaturation during F-T cycles [23,24]. However, there are still some difficulties in the isolation, purification, and identification of novel AFPTs, and their effective and fundamental action still needs to be further explored.

Protein glycosylation has long been recognized as a heterogeneous post-translational modification, contributing to greater protein diversity compared to other post-translational alterations. This diversity significantly influences protein function and exerts profound effects on various biological processes [25]. Deep-sea bony fish are known to produce natural products like antifreeze glycoprotein (AFGP) and antifreeze glycopeptide. A typical AFGP is categorized as a mucin-type glycoprotein containing a tripeptide repeat (*n* = 4–55) of alanine–threonine–alanine (Ala-Thr-Ala), in which the hydroxyl group of threonine is glycosylated by the disaccharide β-D-galactosyl-(1,3)-α-N-acetyl-D-galactosamine [26]. AFGP8 (*n* = 4) has gained attention in the fields of cryopreservation due to its ice recrystallization inhibition activity [27]. Furthermore, analogues of antifreeze glycopeptides, synthesized through non-enzymatic glycosylation methods, demonstrated the ability to preserve cell integrity under low-temperature stress by inhibiting the leakage of intracellular proteins and nucleic acids and by preventing declines in β-galactosidase and lactate dehydrogenase activities, ultimately safeguarding cellular health and sustaining metabolic activity [28]. However, the limited availability of AFGP, coupled with the high costs of purification, restricts their utilization in aquatic products [29].

## 3. Model Action of AFPs and AFPTs

### 3.1. Thermal Hysteresis Activity (THA)

**Figure 1:**
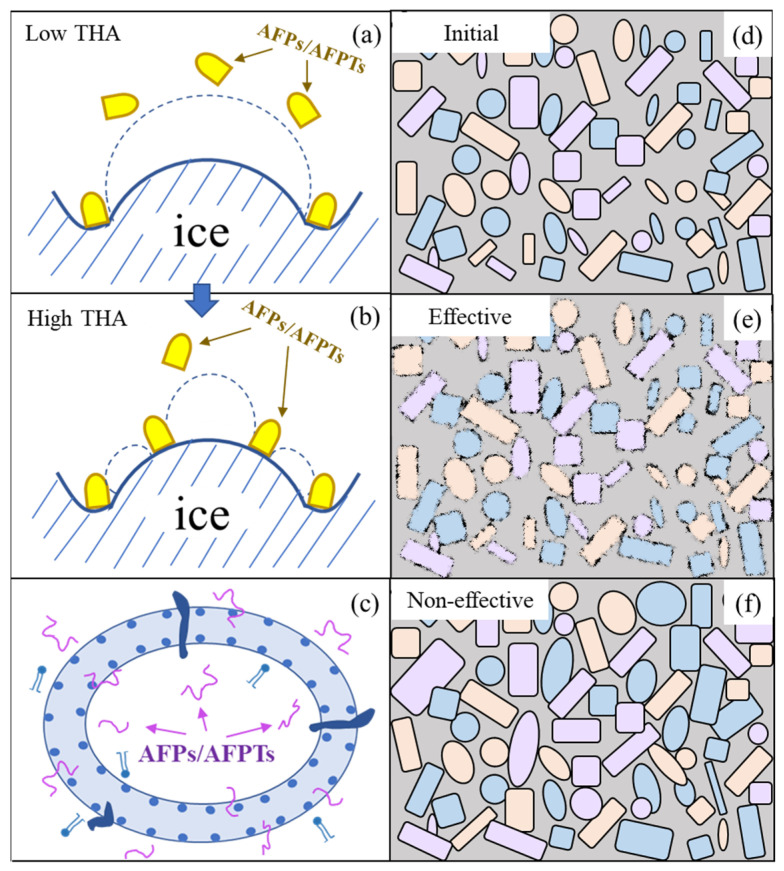
The three activities of antifreeze proteins [28,33,34]. (**a**,**b**) The binding model of the antifreeze protein and ice crystal; (**c**) antifreeze peptides interact with cell membranes; (**d**–**f**) the model of recrystallization inhibition activity. Note: AFPs: antifreeze proteins, AFPTs: antifreeze peptides, and THA: thermal hysteresis.

THA serves as a characteristic measure for AFPs, with it expressing the interaction between AFPs and ice crystals. It illustrates the differences between freezing and melting temperatures (Figure 1a,b). AFP prevents further growth effectively by binding to the surface of the ice crystals. Simultaneously, the adsorption orientation of AFP exerts an impact on the number and coverage rate of protein molecules on the ice crystal surface, thereby giving rise to the THA phenomenon [30]. The strong binding of AFPs to ice entails specific receptor–ligand interaction, involving key forces such as hydrogen bonding, hydrophobic interactions, and van der Waals forces [31]. Additionally, various physicochemical factors, including species diversity, ice mass fraction in samples, and the binding affinity of AFPs with cell membranes, have been shown to impact THA indicators [32,33]. In the case of fish-derived AFPs, the maximum THA value approaches 1 °C. Moreover, in comparison to insect-derived AFPs, fish-derived AFPs exhibit a faster cooling rate, leading to a reduction in apparent THA. As a result, the larger ice crystals tend to initiate crystal growth at higher temperatures [33].

### 3.2. Inhibition of Ice Recrystallization

Ice recrystallization is a thermodynamic process that leads to alterations in the physical properties of ice crystals involving their number, size, shape, orientation, etc. This undesirable phenomenon is mainly generated by three mechanisms, including accretion, migration, and surface isomass [35]. The incorporation of AFPs/AFPTs functions to modulate ice crystals, thereby inhibiting their aggregation and facilitating the formation of uniform and homogeneous ice crystals (Figure 1d–f). Under the action of hydrogen bonding, hydrophobic interactions and van der Waals force, ice crystals can be regulated to reduce the mechanical damage toward organisms [7]. Typically, a minute amount of AFPs can exhibit a pronounced recrystallization inhibitory effect. AFP III, in particular, retains its recrystallization inhibition activity regardless of pH or typical process parameters such as temperature or pressure, rendering it a promising application compound [36]. Moreover, the recrystallization process of frozen sucrose solution can be inhibited to a certain extent by using an antifreeze protein and hydrophilic colloid mixture, and the effect is better than that of a single application [37].

### 3.3. Membrane Protection

Cryodamage to cell structures during freezing is a multifaceted issue encompassing the depolymerization of cell walls, the rupture of cell membranes, alterations in osmotic pressure, etc. The formation of ice crystals within intracellular and extracellular regions during freezing or supercooling can lead to mechanical injury and cold stress, hastening the onset of cell apoptosis, particularly when employing slow freezing methods [38]. Previous research has affirmed that fish-derived AFPs, particularly those derived from fish scale hydrolysates, can interact with phospholipid groups to elevate the phase transition temperature of cell membranes. This, in turn, mitigates ice crystal-induced damage to the cell membrane, preserves intracellular and extracellular ion balance, and ultimately enhances antifreeze capabilities. Moreover, it protects cell membranes from the adverse effects of low temperatures (Figure 1c) [38,39].

### 3.4. “Hydrophilic-Complementary” Model

**Figure 2:**
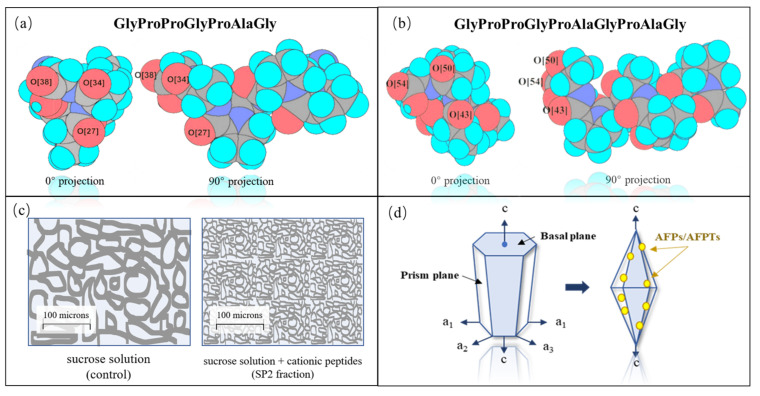
The mechanism of action of antifreeze peptides [7,18,22]. (**a**,**b**) Hydrophilic and “complementary-interaction” model between different antifreeze peptides and the ice layer surface; (**c**) ice crystal inhibition by fish gelation peptides; (**d**) changes in the ice crystal growth trajectory in the “adsorption-inhibition” theory. Note: AFPs: antifreeze proteins; AFPTs: antifreeze peptides.

The molecular structure of AFPs typically exhibits amino acid sequence characteristics of active molecules, featuring the presence of Gly-Pro tripeptide repeat sequences and structural motifs, such as GTPG- and GPP(OH)G. In combination with molecular dynamics simulation technology, a theoretical model was established to elucidate the surface interaction between AFPTs and ice-structured molecules. The mechanism of action between AFPTs and ice-structured molecules was elucidated, indicating a mode of “hydrophilic-complementary” interaction (Figure 2a,b) [18]. Specifically, gelatin peptides form an oxygen triplet plane at the C-terminus, exhibiting an oxygen–oxygen distance similar to that observed in ice nuclei. This oxygen triplet plane engages in hydrogen bonding with the prismatic facets of the ice core, thereby producing a freezing-inhibiting effect (Figure 2c) [22]. Subsequent investigations have confirmed the mechanism through which gelatin peptides inhibit ice crystal growth. It comprises three key steps: (i) a non-specific electrostatic interaction between the cationic peptide and the negatively charged ice surface; (ii) optimal formation of hydrogen bonds with an oxygen–oxygen lattice on the ice surface; and (iii) electrostatic and hydrogen bond establishment, partially non-polar, as well as environmentally stable peptide–ice crystal complexes in proximity with the hydrophobic regions of the peptides [18].

### 3.5. “Adsorption-Inhibition” Theory

The ‘adsorption-inhibition’ theory was also proposed and subsequently confirmed that in an equilibrium solution lacking AFPs/AFPTs, the formation of ice crystals and their subsequent crystallization nearly coincide with the temperature drop. Ice crystals grow from the melting point along the a-axis in a hexagonal or circular pattern (Figure 2d). However, in the presence of AFPTs, interactions between ice and peptides result in a delay in the crystallization process until the temperature decreases sufficiently to overcome the influence of AFPTs. In this context, ice crystals within the hysteresis range expand along the c-axis and exhibit a modified morphology, typically adopting a bipyramidal shape [40,41].

## 4. Freezing Protection of Aquatic Products by AFPs and AFPTs

### 4.1. Origin Muscle and Fillets

The effects of AFPs and AFPTs on the quality of aquatic products and their modes of action have been illustrated (Table 1). Hydrolysis from fish skin and fish scales, rich in collagen or gelatin functional properties, contained low molecular weight peptides [16,42]. They undergo hydrolysis or enzymatic hydrolysis to yield small-molecule AFPs or AFPTs. Generally, these AFPTs have molecular weights below 2000~3000 Da with specific amino acid residues, such as glycine and glutamic acid [17]. In a previous study, low molecular weight (<3000 kDa) fractions assumed a conspicuously preservative effect, with them having a higher THA value, fewer ice nuclei, and more prominent recrystallization inhibition activity [43]. Their antifreeze effects were comparable to commercial antifreeze agents when used in concentrations ranging from approximately 0.1% to 8% [44,45]. Some of the marine or freshwater fish protein, e.g., large yellow croaker and silver carp meat, has been used for hydrolysis [46,47]. Based on the origin of these AFP and AFPT sources, their application on the aquatic products proved more compatible compared with others. To identify the antifreeze properties of these hydrolysates, the F-T cycle procedure was applied to evaluate the protein and lipid oxidation degree of muscle-rich products, such as fish/shrimp muscle or scallop adductor muscle [16,17,48]. Most protein oxidation indices, including sulfhydryl content, carbonyl content, salt-solubility, Ca^2+^-ATPase activity, and MP structure changes, have been found to demonstrate effective antifreeze properties by scavenging vitro free radicals and inhibiting ice crystal transformation [46,49]. For instance, a 0.1% (*w*/*v*) herring skin type I AFP tested for preventing the frozen denaturation of red sea bream cubes and largemouth bass fillets by effectively inhibiting the passive impact of water–ice phase transition, such as freezing point rise, ice crystal shape/growth, water holding capacity reduction, and water mobility/distribution [48,50]. More than that, the herring skin type I AFP prevented MP oxidation and aggregation from protein degradation to maintain its own structural completion, water affinity, and thermal stability by reducing the freezing point and generating thermal hysteresis [44]. Lipid oxidation can promote the deterioration of protein-rich aquatic products. Two varieties of sturgeon skin gelatin hydrolysates have been certified to scavenge activity against DPPH, ABTS, and hydroxyl radicals and reduce lipid peroxide alondialdehyde formation, the formation of free water, and its mechanical damage [23,24,51]. Therefore, several AFPs and AFPTs based on aquatic processing by-products have been prepared recently.

### 4.2. Mince and Surimi Products

With the development of intensive processing of aquatic protein raw materials, more and more resources have been developed and utilized as fish mince- or surimi-based products, which should be applied to cryopreservation technology for long-term preservation. In particular, with the rapid development of 3D printing, as a novel molding technology, the storage stability of fish meat-based ink has become one of the most conspicuous research hotspots [52]. As shown in Table 2, over prolonged periods of frozen storage or during freeze–thaw cycles, these AFPTs elevate the salt-soluble protein content, total sulfhydryl content, Ca^2+^-ATPase activity, and water-holding capacity of fish mince- and surimi-based products [53]. Simultaneously, they reduced surface hydrophobicity, carbonyl content, disulfide bond content, and exhibited strong protection of myosin heavy chains (globular head domain). Lowering the size of ice crystals to reduce mechanical damage was one major mechanism, which can significantly enhance the storage stability of the gel properties of surimi [16]. Protein hydrolysates have been documented to have the capacity to delay the exposure of hydrophobic amino acids and sulfhydryl oxidation of proteins within aquatic products during freezing and frozen storage. Furthermore, the peptides were good at augmenting non-covalent bond interactions with proteins to improve gel properties, especially utilizing the synergistic effects of hydrophilic and hydrophobic amino acid residues [43]. In addition to their antioxidant properties, sliver carp scale AFPTs played an energetic role in improving the rheological and textural properties and reserving the shape of 3D-printed grass carp or sliver carp surimi after F-T cycles by inhibiting ice crystal growth in surimi, preventing denaturation and juice loss of the protein. [52,54]. Hence, the utilization of fish deep-processing by-products in the production of surimi-based products has gained increasing attention, particularly regarding the concurrent application of homologous AFPs/AFPTs and surimi-related fabricated products.

## 5. Conclusions

The quality deterioration of aquatic products is primarily driven by a complex interplay of factors, including mechanical damage, protein denaturation, and drying loss during frozen storage. Aquatic products with high protein content undergo alterations during freezing, frozen storage, and thawing, resulting in diminished water-holding capacity, increased thawing losses and cooking losses, and various changes in solubility, surface hydrophobicity, Ca^2+^-ATPase activity, and denaturation enthalpy. Conventional commercial cryoprotectants have been employed to mitigate these quality issues; however, their uses are constrained by regulatory and health considerations. Recent developments have focused on natural antifreeze proteins and peptides for aquatic raw materials and deeply processed products. Nonetheless, while significant progress has been made, a substantial gap remains between current research findings and industrial-scale production. While some studies have targeted the utilization of aquatic product processing by-products like fish skin and fish scales, as well as specific plant-based proteins, future efforts will center on the investigation of novel processing by-products and the development of innovative engineering technologies for both plant and animal sources.

## Figures and Tables

**Table 1:** Application and mechanism of aquatic resource AFPs/AFPTs in aquatic muscle and fillets.

AFPs/AFPTs Resource	Application	Treatment	Concentration	Function	Mechanism	References
Large yellow croaker (*Pseudosciaena crocea*) meat hydrolysates	Fresh turbot (*Scophthalmus maximus*) cubes	The F-T cycles (stored at −20 °C for 1 day and thawed at 4 °C for 12 h and ended when the core temperature reached 0–4 °C) three times	0.5, 1.0, 2.0 mg/mL	Inhibited ice crystal growth and muscle fiber damage and decreased sulfhydryl content and Ca^2+^-ATPase activity	Reduced MP oxidation caused by ice crystals	[46,49]
Squid skin (*Dosidicus gigas*) collagen hydrolysates	Shrimp (*Penaeus penicillatus*) muscle	The F-T cycles (frozen at −25 °C for 24 h and thawed at 4 °C) 2, 6, 10 and 14 times	1% (*w*/*w*)	Inhibited effects on the denaturation and structural changes of myofibrillar protein; partially retained the ability to bind water; and offered protection for mechanical injury caused by ice crystals	Retarded protein oxidation	[17]
Tilapia (*Oreochromis mossambicus*) skin collagen hydrolysate	Scallop (*Chlamys farreri*) adductor	Snap frozen at −75 °C for 2 h and stored at −18 °C for 8 weeks	0.5, 1, 2, 3 g/100 g	Increased thermal hysteresis activity, exhibited a higher salt soluble protein concentration, total sulfhydryl content, Ca^2+^-ATPase activity, and water-holding capacity	Reduced MP denaturation	[16]
Herring (*Clupea harengus*) skin type I AFP	Red sea bream meat pieces (*Pagrosomus major*)	The F-T cycles (frozen at −20 °C for 24 h and thawed at 4 °C for 12 h) once, thrice, and five times	0.1% (*w*/*v*)	Improved thermal stability and viscoelasticity and restricted water mobility and distribution	Inhibited MP oxidation and aggregation	[50]
	Largemouth bass (*Micropterus salmoides*) fillets	Soaked at 4 °C for 12 h, frozen at −20 °C for 24 h, and thawed at 4 °C overnight	0.05, 0.08, 0.1, 0.5% (*w*/*v*)	Inhibited ice crystal growth, modified ice crystal sharpness, increased thermal stability, and improved the spatial network structure of protein	Prevented MP oxidation and aggregation	[48]
		The F-T cycles (frozen at −20 °C for 24 h and thawed at 4 °C for 12 h) 3 times	0.1% (*w*/*v*)	Reduced the freezing point and generated thermal hysteresis	Delayed MP degradation	[44]
Silver carp (*Hypophthalmichthys molitrix*) fin hydrolysates	Bighead carp (*Hypophthalmichthys nobilis*) fillets	The F-T cycles (stored at −18 °C for 1 week and thawed at 4 °C until the core temperature to 0 °C) 0, 2, 4, and 6 times	2% (*w*/*v*)	Exhibited in vitro scavenging activity (ABTS radicals) and chelating activity to ferrous ions and inhibited the formation of carbonyls and disulfide bonds and the loss of Ca^2+^-ATPase activity	Reduced protein/lipid oxidation and degradation	[42]
Beluga sturgeon (*Huso huso*) skin gelatin hydrolysates	Freshwater crayfish (*Astacus leptodactylus*) muscle	The F-T cycles (stored at −18 °C) 6 times	8% (*w*/*v*)	Reduced secondary lipid oxidation and the loss of in sulfhydryl groups and Ca^2+^-ATPase activity; scavenged free radicals and chelated ferrous ions	Inhibited myosin heavy chain denaturation and impeded lipid oxidation	[51]

**Table 2:** Application and mechanism of aquatic resource AFPs/AFPTs in aquatic mince and surimi.

AFPs/AFPTs Resource	Application	Treatment	Concentration	Function	Mechanism	References
Silver carp (*Hypophthalmichthys molitrix*) scale hydrolysates	Grass carp (*Ctenopharyngodon idella*) surimi	The F-T cycles (stored at −20 °C for 72 h and thawed at 25 °C for 1 h) 5 times	2, 4, 6, 8%	Delayed sulfhydryl oxidation, the carbonylation of amino acids, and the exposure of hydrophobic amino acids by inhibiting ice crystal growth	Delayed MP degradation	[55]
	Grass/Sliver carp surimi ink for 3D printing	The F-T cycles (stored at −20 °C for 5 days and thawed at 25 °C for 4 h) 4 times	1, 2, 4, 6, 8% (*w*/*w*)	Protected the rheological properties of surimi ink (viscosity, τ0, shear-thinning characteristics, viscosity recovery, temperature recovery, and modulus)	Provided more interaction points for protein	[52,54]
Sliver carp meat hydrolysates	Sliver carp surimi	The F-T cycles (stored at −25 ± 1 °C for 24 h and thawed at 4 ± 1 °C for 12 h) 6 times	2 g/100 g	Displayed lower salt-soluble protein extractability loss, less actomyosin Ca^2+^-ATPase activity decrease, and unfrozen water content decrease	Protected against MP denaturation; absorbed the ice surface; and inhibited ice crystallization	[47]
Sliver carp by-product (fish meat leftovers on bones and heads) hydrolysates			2, 4, 6 g/100 g	Displayed higher actomyosin extractability, Ca^2+^-ATPase activity, and, correspondingly, lower surface hydrophobicity of actomyosin; presented comparable textures for gels	Decreased protein denaturation/aggregation and improved gel-forming capacity	[56]
Sliver carp protein hydrolysates	Unwashed sliver carp surimi	The F-T cycles (stored at −18/−60 °C for 12 h and thawed at 4 °C for 12 h) 3 and 6 times	0.4, 0.8 g/100 mL MP solution	Alleviated carbonyl content for MP, decreased free sulfhydryl content, and maintained the protein bands’ stability; slowed down lipid peroxide malondialdehyde formation and flavor compound change rates in unwashed surimi; and interacted with the proteins to alleviate the water loss and structural collapse of the gel	Inhibited MP oxidation, aggregation and denaturation, and lipid oxidation	[57]
Bighead carp (*Hypophthalmichthys nobilis*) gill protein hydrolysates	Silver carp surimi	Frozen at −18 °C for 4 months	1, 2% (*w*/*w*)	Improved the texture, and the properties reduced the decrease in salt-soluble protein content and Ca^2+^-ATPase activity	Scavenged free radicals and chelate metal ions	[58]
*Takifugu obscurus* skin	Grass carp surimi	The F-T cycles (frozen at −25 °C for 3 days and thawed at 25 °C water bath) 5 times	2, 4, 6, 8% (*w*/*w*)	Inhibited the growth and recrystallization of ice crystals, prevented protein denaturation, and maintained the spatial structure and water retention ability of proteins	Inhibited protein freezing-induced oxidation	[1,59]
Pacific hake (*Merluccius productus*) protein hydrolysates	Pacific cod (*Gadus macrocephalus*) mince and fish ball	The F-T cycles (stored at −25 °C for 18 h and thawed at 4 °C for 6 h) 6 times	2, 4, 6, 8% (*w*/*w*)	Improved water-holding capacity and thermal stability, retarded salt extractable protein degeneration, and enhanced flavor and texture	Indicated the protein stabilization and cryoprotective effect	[45]
Amur sturgeon (*Acipenser schrenckii*) skin gelatin hydrolysate	Japanese sea bass (*Lateolabrax japonicas*) unwashed mince	The F-T cycles (frozen at −18 °C for 20 h and thawed at 4 °C for 4 h) 3 and 6 times	8% (*w*/*w*)	Stabilized the water associated with myofibrils, retarded protein carbonyl formation, and caused lower loss of sulfhydryl content	Protected against protein and lipid oxidation	[60]
Amur sturgeon skin gelatin tetrapeptide (Pro-Ala-Gly-Tyr)	Japanese sea bass mince	The F-T cycles (frozen at −14 °C for 18 h and thawed at 4 °C for 6 h) 6 times	25, 50, 100, 200 ppm	Scavenged activity against DPPH, ABTS, and hydroxyl radicals, prevented lipid oxidation, influenced water distribution, and decreased myosin and actin denaturation	Showed antioxidative and cryoprotective effects	[24]
Rainbow trout (*Oncorhynchus mykiss*) processing by-product hydrolysates	Asian seabass (*Lates calcarifer*) mince	Pre-frozen at −18 °C for 24 h and underwent F-T cycles (frozen at −18 °C for 18 h and thawed at 4 °C for 6 h) 6 times	4, 8%	Decreased the loss of total sulfhydryl groups and protein solubility and protein carbonyl formation	Maintained the thermal properties and water bonding ability of the MP	[19]
Surimi processing by-products (fish head, skin, scale, bone, dark muscle, etc.) hydrolysates	Silver carp surimi	Surimi paste frozen at −18 °C 3 months	2%	Retarded sulfhydryl oxidation, carbonylation, myosin denaturation, and the exposure of hydrophobic amino acids and improved the gelation properties and water-holding capacity of gels	Chemical antioxidants and cryoprotectants	[61]

## Data Availability

No new data were created or analyzed in this study. Data sharing is not applicable to this article.
